# Severe myocarditis due to influenza A(H1N1)pdm09 viral infection in a young woman successfully treated with intravenous zanamivir: A case report

**DOI:** 10.1002/ccr3.2499

**Published:** 2019-10-21

**Authors:** Maria Mazzitelli, Eugenio Garofalo, Andrea Bruni, Giorgio Settimo Barreca, Angela Quirino, Aida Giancotti, Francesca Serapide, Ciro Indolfi, Giovanni Matera, Paolo Navalesi, Enrico Maria Trecarichi, Carlo Torti, Federico Longhini, The IMAGES (Integrated MAnaGEment of Sepsis) Group, Cinzia Peronace, Vincenzo Pisani, Chiara Costa, Giuseppe Greco, Valentina La Gamba, Vincenzo Scaglione, Eugenio Biamonte, Vincenzo Brescia, Brunella De Leonardis, Abdalla Karim, Giuseppina Cimino, Paola La Torre, Antonio Gemelli, Francesco Antonio Tropea, Francesco Picicco, Luigia Gallo

**Affiliations:** ^1^ Department of Medical and Surgical Sciences, Infectious and Tropical Diseases Unit “Magna Graecia” University Catanzaro Italy; ^2^ Department of Medical and Surgical Sciences Unit of Intensive Care, “Magna Graecia” University Catanzaro Italy; ^3^ Department of Health Sciences Unit of Clinical Microbiology “Magna Graecia” University Catanzaro Italy; ^4^ Department of Medical and Surgical Sciences Division of cardiology “Magna Graecia” University Catanzaro Italy

**Keywords:** influenza A(H1N1)pdm09 virus, intravenous zanamivir, myocarditis

## Abstract

In patients with influenza‐related myocarditis, prompt diagnosis and treatment are important. Intravenous zanamivir can be an alternative to oral oseltamivir, especially in severe cases and when drug intestinal malabsorption is suspected or proven.

## Graphical abstract

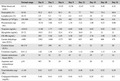

## 1 INTRODUCTION

Influenza virus may affect myocardial muscle, producing severe myocarditis, a clinical condition with a high mortality rate. We report a case of a 23‐year‐old woman diagnosed with acute A(H1N1)pdm09‐related myocarditis. After a timely diagnosis and prompt intravenous treatment with zanamivir, the patient experienced a full clinical response.

Influenza causes every year a great burden of diseases worldwide, with a high associated mortality, especially among fragile patients. Influenza may involve organs other than lungs, such as heart with exacerbation of underlying conditions (eg, ischemic heart disease), myocarditis, and/or pericarditis.^1^

Myocarditis is an acute inflammation of myocardium, which is diagnosed in approximately 0.4%‐13% of hospitalized adult with documented influenza.^1^ As for epidemiological and clinical characteristics, patients with influenza‐related myocarditis are predominantly young (mean age 33.2 years) and female (63%).^2^ Influenza‐associated myocarditis spans a wide spectrum of clinical pictures, from asymptomatic to severe and fulminant disease, with hemodynamic shock, requiring high dosage of catecholamine and extracorporeal circulatory support.^1^ This condition is characterized by a high mortality (up to 27%).^2^

In patients hospitalized for severe or progressive pandemic influenza A(H1N1)pdm09 infection, early treatment with oseltamivir, a neuraminidase inhibitor, is strongly recommended.^3^ Although a very limited case series showed that oseltamivir is well absorbed when given through nasogastric feeding tube,^4^ oseltamivir cannot be considered an optimal empiric in such cases for still persistent pharmacokinetic constrains. Moreover, patients requiring mechanical ventilation may be affected by defective enteral drug absorption, which may further harm oseltamivir absorption.^5^ Lastly, resistance‐associated mutations of influenza A(H1N1)pdm09 virus to oseltamivir could limit its activity,^6^ especially in cases when bioavailability maybe not guaranteed.

In the last few months, intravenous (IV) zanamivir (600 mg twice daily) became available for treatment of patients with severe influenza.

To date, data on outcome of adult patients with influenza‐related myocarditis treated with IV zanamivir are scarce. To the best of our knowledge, only Jahns et al^5^ recently reported two cases of acute myocarditis in patients with influenza successfully treated with IV zanamivir. Herein, we report a case of an influenza A(H1N1)pdm09 virus, complicated by severe myocarditis, timely treated with IV zanamivir available through a compassionate use program.

## 2 CASE REPORT

During influenza season 2017‐2018, a 23‐year‐old Caucasian woman, with partial trisomy 1q from DE NOVO translocation 1:8,^7^ was admitted to a peripheral hospital and immediately transferred to our tertiary‐level intensive care unit (ICU) for acute respiratory distress syndrome (ARDS), to be evaluated for extracorporeal membrane oxygenation (ECMO) support.

Five days before hospital admission, the patient reported onset of fever, cough, and shortness of breath. At admission and before starting empiric antimicrobial therapy (ceftazidime 2 g IV three times daily, levofloxacin 500 mg IV twice daily, and vancomycin 2 g IV daily), we immediately performed routine microbiological cultures (blood, urine, flu testing, and surveillance for multidrug‐resistant bacteria).

Protective mechanical ventilation was instituted in volume‐controlled mode, in association with continuous IV infusion of neuromuscular blocking agents (cisatracurium besylate 1‐2 mcg*kg/min). Arterial blood gases (ABGs) showed a moderate alteration of gas exchange (arterial partial pressure to inspired fraction of oxygen [PaO_2_/FiO_2_] of 168 mm Hg) not requiring ECMO support.

Because of persistent hypotension (ie, mean arterial pressure <65 mm Hg) despite fluid resuscitation, a pulmonary artery catheter (Swan‐Ganz CCOmbo V, Edwards Lifesciences LLC) was inserted for hemodynamic assessment, and continuous infusion of norepinephrine (0.7 mcg*kg/min) and dobutamine (5 mcg*kg/min) was started. The body temperature was 38.4°C. Sequential Organ Failure Assessment (SOFA) score was 8.

In addition to high inflammatory indexes, we found signs of acute liver injury, a mild increase in blood creatinine levels, and an abnormal increase of cardiac and muscular enzymes. Blood test results along the ICU admission are shown in Table 1. No electrocardiogram alterations were found, while cardiac ultrasound revealed transient wall thickening, reduction of wall motion with severe left ventricular ejection fraction (LVEF) of 30%, and mild (~3 mm) pericardial effusion.

**Table 1:** Blood test along with ICU admission

	Normal range	Day 0	Day 3	Day 6	Day 9	Day 12	Day 15	Day 18	Day 21
White blood cell (n/µL)	4.5‐11	16.17	11.8	15.55	12.58	12.47	11.93	8.49	6.52
Neutrophils (%)	45‐62	92.5	82.5	85.1	91	77	79.5	75.4	68.6
Lymphocytes (%)	16‐33	2.9	5.3	7.3	4.4	13.5	10	13	17.8
Platelets (n*10^3^/µL)	150‐400	181	103	224	402	551	551	464	416
Procalcitonin (ng/mL)	<0.2	19.32	3.66	0.37	1.34	3.21	1.08	0.23	0.08
Troponin (ng/mL)	<0.014	11.00	1.77	3.85	0.69	0.06	0.03	0.02	0.01
Myoglobin (ng/mL)	25‐72	1013	23.3	22.4	47.4	26.9	21	21	21
CK‐Mb (ng/mL)	<3.61	203	5.56	2.45	4.28	3.67	2.76	2.98	2.40
Lactate dehydrogenase (IU/L)	<600	1562	1440	1173	895	653	662	606	479
Creatine kinase (IU/L)	60‐174	5197	290	44	251	38	22	25	20
Creatinine (mg/dL)	0.8‐1.2	1.62	1.56	1.37	1.20	1.12	1.08	1.13	1.16
Alanine aminotransferase (IU/L)	≤34	54	32	23	45	15	19	17	14
Aspartate aminotransferase (IU/L)	≤34	467	78	24	56	15	20	21	15
Total bilirubin (mg/dL)	<1.40	0.61	0.57	0.86	0.73	0.48	0.39	0.52	0.39
Conjugated bilirubin (mg/dL)	<0.40	0.40	0.41	0.53	0.44	0.33	0.25	0.2	0.2

We therefore postulated three main differential diagnosis as follows: (a) myocarditis; (b) direct myocardial damage due to severe septic shock (ie, Takotsubo stress cardiomyopathy); and (c) sepsis‐induced myocardial depression. So, following criteria set up by European Society of Cardiology (ESC) Working Group on Myocardial and Pericardial Diseases,^8^ we made diagnosis of myocarditis, with no need to perform EMB. Indeed, our patient presented clinical criteria and two diagnostic criteria (altered myocardiocytolysis markers and functional/structural abnormalities on cardiac ultrasound). Furthermore, chest high‐resolution computed tomography (HRCT) showed a diffuse ground glass aspect with pulmonary consolidations.

All microbiological cultures came back as negative, but polymerase chain reaction (PCR) performed on nasopharyngeal swab (Biofire^®^ FilmArray^®^ Respiratory Panel, BioMèrieux diagnostics) came back as positive for influenza A(H1N1)pdm09 virus. After obtaining urgent local ethical committee approval, IV zanamivir (600 mg twice daily) was administered for compassionate use, the same day of ICU admission (Day 0), in adherence with the European Medical Agency (EMA) recommendations.^9^

On day 3 from ICU admission and zanamivir administration, a follow‐up cardiac ultrasound showed a general improvement of myocardial wall motion and indexes (LVEF 40%). Therefore, catecholamines dosing was progressively reduced and stopped on day 5. Cardiac enzymes also progressively improved, as well as liver function tests.

On day 10, ventilator‐associated pneumonia was diagnosed, and a bronco‐aspirate cultural exam showed extensively drug‐resistant *Acinetobacter baumannii* infection (susceptible only to colistin) with new lung infiltrates at chest X‐ray.^10^ So, colistin (4.5 MU IV twice daily and 1.5 MU per aerosol twice daily), rifampicin 600 mg IV daily, and meropenem 2 g IV three times daily for 14 days were prescribed.

On day 12, nasal‐pharyngeal swab results became negative for influenza A(H1N1)pdm09 virus, and zanamivir was withdrawn. On day 14, the patient recovered from hypoxemic acute respiratory failure (PaO_2_/FiO_2_ 236 mm Hg with a positive end‐expiratory pressure of 8 cmH_2_O), and she was weaned off from invasive mechanical ventilation through noninvasive ventilation (NIV).^11^ On day 16, NIV was stopped, and high‐flow oxygen through nasal cannula was started to proceed with weaning and a progressive respiratory muscle unload.^12^ Patient was discharged from ICU on day 21, with complete resolution of the disease and improvement of blood tests. On day 34, the patient was discharged to home. At 60‐day follow‐up, both echocardiography and blood tests were completely returned within normal ranges.

## 3 DISCUSSION

During influenza season, particular awareness is necessary on possible complications of influenza virus. Among 58 patients with influenza A(H1N1)pdm09 virus infection, the prevalence of influenza‐related myocarditis was reported to be around 62%.^2^, ^13^ Myocarditis diagnosis is generally clinical, based on a combination of symptoms, elevated cardiac enzymes, and echocardiographic findings.^1^ Furthermore, most patients presented nonspecific abnormalities at electrocardiogram, such as ST elevation (34%) and inverted T waves (24%).^2^, ^13^ Nowadays, almost 50 cases of influenza‐related myocarditis are described in the literature; among these cases, in 70% of patients, influenza A(H1N1)pdm09 virus was isolated.^1^

During pandemic influenza seasons, it is crucial to routinely perform flu swabs to every patient admitted for respiratory disease. Moreover, surveillance and high level of attention for influenza‐related complications are required. Beyond a timely diagnosis, a prompt prescription of an adequate antiviral treatment is fundamental for the clinical outcome. Originality of our case consists in the prompt initiation of IV zanamivir, started just few hours after ICU admission (as soon as influenza A(H1N1)pdm09 virus was detected in the nasopharyngeal swab), with excellent clinical outcome. In individuals who may suffer from immunosuppression (which might be correlated to partial trisomy 1q syndrome in our patient), this proactive approach may be particularly useful.^7^

Our patient was prescribed high supportive measures, such as invasive mechanical ventilation, deep sedation, neuromuscular blocking, and vasoactive agents, affecting gastrointestinal peristalsis and adsorption of drugs administrated via nasogastric feeding tube. So, the recommended first line treatment, oseltamivir administered orally, could have been less effective than zanamivir for pharmacokinetic constrains.^14^ Good bioavailability of oseltamivir administrated orally via nasogastric tube was not extensively demonstrated.^4^ Moreover, oseltamivir may be less effective than zanamivir for H1N1 strains with resistance mutations. Therefore, IV zanamivir as empiric treatment in patients with severe clinical conditions lets overcome limitations due to resistant strains and impairment of intestinal absorption. Moreover, compared to zanamivir, oseltamivir may prolong viral excretion and symptoms in case of infections due to H1N1 influenza strains harboring H274Y mutation.^15^ Therefore, oseltamivir cannot be considered to be a good empiric treatment in any cases, while IV zanamivir may allow to treat strains of influenza resistant to oseltamivir or patients with enteral malabsorption. The use of IV zanamivir was already shown to be effective in the treatment of patients with influenza A(H1N1)pdm09‐related pneumonia.^14^

Recently, Jahns et al^5^ reported two cases of patients treated with IV zanamivir for influenza‐related myocarditis and enteral malabsorption, ascertained with a paracetamol absorption test. These two patients had more severe damage of heart function than ours, so extracorporeal life support for cardiogenic shock was necessary.^5^ However, similarly to our case, cardiac function rapidly improved, with an almost complete resolution of the heart impairment.^5^

Both patients reported by Jahns et al^5^ and our case are characterized a major limitation. Indeed, endomyocardial biopsy (EMB) was not performed, in our case to avoid further complications in a patient who was already in critical condition. Moreover, clinical conditions improved promptly after IV zanamivir, not requiring further tests. By contrast, as previously shown by Ito et al,^16^ influenza A(H1N1)pdm09 virus would probably have not been detected in myocardial cells since sensitivity of this exam is suboptimal. This is mainly due to lower virus burden in the myocardium, compared to the respiratory tract.^17^ Although EMB remains the gold standard for diagnosis of myocarditis, it is used infrequently,^8^ and other criteria have been listed and validated by ESC as reliable to establish a diagnosis.

## 4 CONCLUSIONS

During influenza seasons, particular surveillance strategies are required to promptly diagnose influenza A(H1N1)pdm09 virus infection and its possible complications, such as myocarditis. This is especially necessary for those patients who are more likely to develop severe complications, such as pregnant women, elderly people, and patients with comorbidities impairing immune systems. As for the latter, genetic disorders similar to that of our patient have been associated with a spectrum of immunological abnormalities.^7^

Prompt initiation of IV zanamivir was successful in our patient, despite her immunocompromised status, the respiratory diseases, and the severe myocarditis. To date, data are still very limited and further studies with an adequate design are necessary to provide more strong evidences about the ideal management of such patients. Awaiting further and more powerful studies, we believe that our results provide further support to the very recent granting of marketing authorization for IV zanamivir under exceptional circumstances set by EMA.^9^

## CONFLICT OF INTERESTS

The authors declare that they have no competing interests.

## AUTHOR CONTRIBUTIONS

CT, MM, and EMT: participated to care of patients as infectious disease consultant physician, wrote and revised the paper. FL and PN: took care of patients in the ICU department, wrote and revised the paper. CI: participated to care of patients as cardiologist consultant physician and revised the paper. AQ, AG, and GM: participated in the microbiological tests and revised the paper. FS, EG, and AB: participated to care of the patients, collected the date and revised the paper. GSB: performed the molecular tests. All authors approved final version of this manuscript and agreed to be accountable in ensuring that all questions related to the accuracy or integrity of any part of the work are appropriately investigated and resolved.

## ETHICAL APPROVAL

Calabria Region Ethics Committee approved aqueous solution zanamivir use for the patient.

## CONSENT FOR PUBLICATION

Signed consent for publication was obtained.

## CONSENT TO PARTICIPATE

Patient's relatives signed the informed consent modules for treatment and publication of clinical data for research and scientific purposes.

## Data Availability

All data generated or analyzed during this study are included in this paper.
